# Preparation and Characterization of Rare Earth Doped Fluoride Nanoparticles

**DOI:** 10.3390/ma3032053

**Published:** 2010-03-19

**Authors:** Luiz G. Jacobsohn, Courtney J. Kucera, Tiffany L. James, Kevin B. Sprinkle, Jeffrey R. DiMaio, Baris Kokuoz, Basak Yazgan-Kukouz, Timothy A. DeVol, John Ballato

**Affiliations:** 1 Center for Optical Materials Science and Engineering Technologies (COMSET), School of Materials Science and Engineering, Clemson University, Clemson, SC, USA; E-Mails: luiz@clemson.edu (L.G.J.); courtnk@clemson.edu (C.J.K.); tjames3@clemson.edu (T.L.J.); ksprink@clemson.edu (K.B.S.); dimaio@tetramertechnologies.com (J.R.D.); bariskokuoz@gmail.com (B.K.); basakkokuoz@gmail.com (B.Y.K.); 2 Department of Environmental Engineering and Earth Sciences, Clemson University, Clemson, SC, USA; E-Mail: devol@clemson.edu (T.A.D.)

**Keywords:** nanoparticle, core/shell, fluoride, luminescence, rare earth

## Abstract

This paper reviews the synthesis, structure and applications of metal fluoride nanoparticles, with particular focus on rare earth (RE) doped fluoride nanoparticles obtained by our research group. Nanoparticles were produced by precipitation methods using the ligand ammonium di-*n*-octadecyldithiophosphate (ADDP) that allows the growth of shells around a core particle while simultaneously avoiding particle aggregation. Nanoparticles were characterized on their structure, morphology, and luminescent properties. We discuss the synthesis, properties, and application of heavy metal fluorides; specifically LaF_3_:RE and PbF_2_, and group IIA fluorides. Particular attention is given to the synthesis of core/shell nanoparticles, including selectively RE-doped LaF_3_/LaF_3_, and CaF_2_/CaF_2_ core/(multi-)shell nanoparticles, and the CaF_2_-LaF_3_ system.

## 1. Introduction

Nanostructures are an integral part of nature, being present in the environment and living organisms where they play important roles in chemical and biological processes. They are the result of natural processes including combustion, precipitation, and phase segregation. Unsuspectingly, nanomaterials have been used for millennia by medical professionals and craftsmen [1]. For example, silver and gold colloids were used for health treatment, including longevity and antibacterial treatments, and colloidal suspensions were used for cloth coloration. A now popular example of this fact is the Lycurgus cup, a Roman goblet from the 4^th^ century A.D., whose coloration is green or red when observed in reflection or transmission, respectively, due to light scattering induced by metallic nanoparticles dispersed in the glassy matrix [2]. While oblivious to the existence and the mechanisms that lead to the unique properties of nanomaterials but to their credit, these professionals were sometimes able to formulate and rigorously follow precise recipes to fabricate materials with specific properties in mind. This is illustrated by the fabrication of scimitar steel blades where the ubiquitous presence of carbon nanotubes and cementite nanowires forming a nanocomposite led to superior mechanical properties [3].

A thorough scientific investigation of nanomaterials is a relatively recent undertaking. The modern perspective on nanomaterials was perhaps first envisioned and articulated by Feynman in 1959, in his now-famous speech “There’s plenty of room at the bottom” [4], meaning that the control of materials and their properties in the atomic scale comprised a new frontier of opportunity in science and technology. Nevertheless, it took additional *ca*. 30 years for the scientific community to massively focus on the many aspects of nanoscience. In the mid-1980’s, Gleiter *et al.* pioneered the systematic investigation of nanostructured materials, coining the terms “nanocrystalline” and “nanocrystal” [5]. Not coincidently, the interest in this new realm in materials science is concomitant to the development of tools to synthesize, manipulate and characterize nanomaterials most effectively [6]. Of particular relevance to this review is the report of intense visible photoluminescence from porous silicon attributed to quantum confinement effects in Si nanocrystals by Canham in 1990 [7] that spurred considerable investigative effort toward the understanding and exploration of light emission from nanostructures. Driven primarily by technological applications, nanophotonics research has largely focused on semiconductor quantum dots, with little attention afforded insulators [8].

Nanoparticles compose a unique class of materials that have reduced dimensions, typically within the range of 1 to 100 nm, and typically are strongly influenced by their surface characteristics and embedding environment. The relative dominance of surface atoms in nanoparticles can be understood when one considers that a spherical nanoparticle composed of 10^4^ atoms (*ca*. 4 nm in diameter) will have about 20% of its atoms in the outermost atomic layer. A more realistic description that considers the surface of a nanoparticle to be the volume of a thin shell, instead of just the outermost atomic layer, will increase this fraction considerably; in a 4 nm diameter nanoparticle with a 0.4 nm thick surface layer nearly 50% of the atoms will compose the surface.

It is also important to consider that a surface commonly differs from bulk characteristics and properties. The loss of the three-dimensional periodicity of the atomic potential at the surface layer will result in changes to the electronic properties. The lack of atoms on one side of the surface that are necessary to counter-balance and fully compensate chemical bonds and charge requirements generates structural modifications like relaxation and reconstruction, and dangling bonds. These changes are known to alter electronic properties and chemical reactivity. Modification of the surface layer can also occur by the presence of contaminants originated in the exposure to the environment (e.g., oxidation), or by interactions with the embedding medium if nanoparticles are dispersed in a liquid (solution) or in a solid host (nanocomposite). For example, a 50% change in the lifetime of Y_2_SiO_5_:Ce nanoparticles prepared by the hydrothermal method was observed when nanoparticles were dispersed in different liquids [9]. Also, surface modification of insulating nanoparticles can lead to enhanced luminescence efficiency as illustrated by the works of Stouwdam and van Veggel [10], and Kömpe *et al.* [11] who reported substantial increase in the quantum yield of rare earth (RE) doped nanoparticles due to surface modifications. The manipulation of the surface characteristics of nanoparticles by organic or inorganic shells is an important topic of research, and in this review we will discuss our results on inorganic shelling on the luminescent properties of RE doped fluoride nanoparticles.

Photonics is the study of all applications of light, including generation, emission, transmission, and amplification, and, among other goals, aims at replacing electric currents by light pulses and beams in fields such as telecommunications and information processing. Metal fluorides are strategic materials in optical and photonic technologies, finding application in lighting, optical amplification, and lasing industries. Luminescent materials also find application in the field of radiation detection as scintillators for medical, scientific, industrial, and security applications. In this case, light is emitted upon impingement of energetic electromagnetic and particle radiation on a phosphor. Some well-known fluoride scintillators like CaF_2_:Eu, BaF_2_, and CeF_3_ are used in high energy physics experiments [12].

Underpinning all these applications are the excellent optical properties of metal fluorides, and particularly the fact that many of them can readily be doped with RE ions and efficiently luminesce. Fluorides are transparent over a wide spectral window. For example, BaF_2_ is transparent from 150 nm to 15 μm, and CaF_2_ from 130 nm to 12 μm [13]. Fluorides also have high band gap wherein allowed electronic transitions of the dopants occur and self-absorption is avoided; the band gap of BaF_2_ and CaF_2_ is 9.1 and 10 eV, respectively [13]. Further, fluorides also posses low phonon energies that minimize the probability for non-radiative transitions. In LaF_3_ and CaF_2_, the effective phonon energy is 43.4 meV (350 cm^-1^) and 40.7 meV (328 cm^-1^), respectively [14,15]. Interestingly, many fluorides have cubic structure and thus are optically isotropic.

Besides the attractive structural and optical characteristics of fluorides, nanoparticles of this family of material have not received as much attention from the scientific community as other optical and optoelectronic nanomaterials like, e.g., semiconductor quantum dots. To date, the most investigated fluoride nanoparticles were LaF_3_ and CeF_3_ with the first papers being published around the year 2000 [16,17,18,19,20,21,22]; other fluorides like BaF_2_ and CaF_2_ received minimal attention [23,24,25,26,27,28]. These nanoparticles were investigated for application in myriad fields, from tribological additives in lubricating oils [16,18] to luminescent properties [19,20,22,23,26,28], when they are doped with REs and corresponding to the topic of this review.

In this review, we report on the results obtained by our research group, focusing on the synthesis and characterization of group IIA fluorides and the heavy metal fluorides LaF_3_ and PbF_2_. Moreover, we also report on an interesting materials strategy that can be explored with nanoparticles that is the core/shell architecture. It is possible to combine the properties of different compounds to achieve enhanced functionalities in core/shells in a unique way. The potential of application of core/shell nanoparticles is as exciting as are the challenges to synthesize them. In this review, we also discuss controlled attempts to obtain core/shell nanoparticles of fluorides, including core/shell nanoparticles composed of different materials.

## 2. Results and Discussion

This section is divided into two parts, the first is related to the heavy metal fluorides LaF_3_:RE and PbF_2_ (core/multi-shell) nanoparticles, and the other to group IIA fluorides and their core/shell structures.

### 2.1. Heavy Metal Fluorides: LaF_3_:RE and PbF_2_

Our research group has carried out extensive investigation of RE-doped core/shell LaF_3_ nanoparticles [29,30,31,32]. In this review, the main results are summarized and the synthesis of these nanoparticles is described in detail in Section 3. The proposed procedure allows the synthesis of core/multi-shell nanoparticles where a given shell may or may not be doped. This has been used to tailor the energy transfer between different dopants, namely Tb^3+^ and Eu^3+^, from zero to partial to total in order to achieve spectral design of these luminescent nanoparticles. By changing the thickness of an undoped shell between the Tb- and Eu-doped shells from 0 to up to 2 nm, it was possible to vary the energy transfer in such a way that the ratio of 540 nm Tb^3+^ peak to that of the 590 nm Eu^3+^ peak decreased from 2.4 to 0.2 [30].

A similar strategy was used to obtain white-light emissions through the combination of different RE dopants, namely Eu^3+^, Tb^3+^, and Tm^3+^ that emit in the red, green and blue, respectively, each doping an individual shell. Precise control of the energy transfer between them through changes in the thickness of the shells allows for these RE dopants that do not share a common excitation to be excited through down-conversion processes. Consequently, the intensity of each of the primary colors can be controlled by the thickness of the shell doped with the corresponding RE responsible for that primary color, and through thickness changes it is possible to navigate within the color coordinate system and tune the emission of the nanoparticles [31].

These unique core/shell structures were also used to investigate kinetic and diffusion effects in optical materials. Heavily Eu^3+^-doped LaF_3_ core nanoparticles single-shelled with undoped LaF_3_ were prepared and thermally treated as a function of temperature and time, and probed by means of photoluminescence measurements. Spectroscopy of the emission and phonon sideband was capable of extracting evidence of the diffusion of Eu^3+^ ions from the core into the shell, particularly because emissions from higher energy manifolds are no longer concentration-quenched. A simplistic one-dimensional diffusion model was employed to extract the effective diffusion coefficient of Eu in LaF_3_, yielding results that are accurate within the order of magnitude in comparison to RE diffusion in CaF_2_ crystals [32].

**Figure 1:**
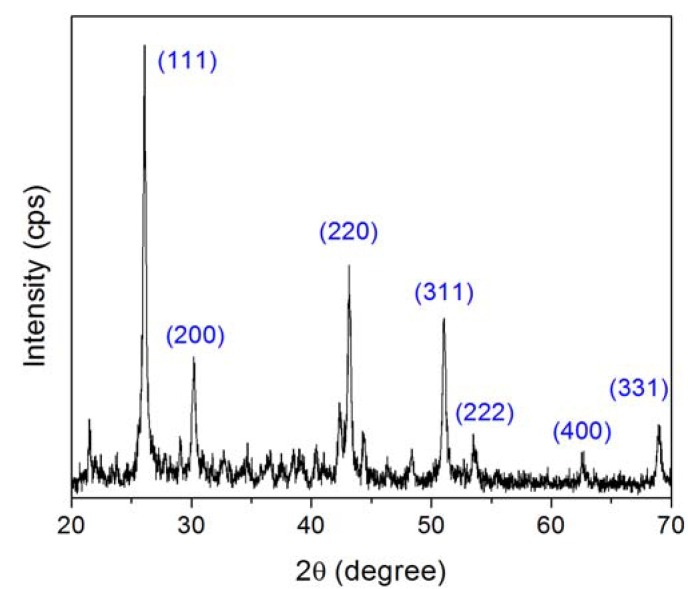
XRD results revealing the structure of PbF_2_ nanoparticles in agreement with JCPDF file 06-0251. Major diffraction peaks corresponding to cubic PbF_2_ are identified; the numerous weak diffraction peaks are originated from the ligand.

Another example of heavy metal fluoride nanoparticle obtained by our group is PbF_2_. Due to its low melting temperature of 824 °C, this material has been used to lower the melting temperature of glasses, and has been incorporated in glasses to increase the index of refraction. Though attractive for radiation detection due to its transparency and high atomic number, this material is known not to scintillate under gamma excitation when doped with REs [33]. Further, heavily Yb-Er co-doped PbF_2_ nanoparticles have been suggested as nanoscale fluorescent thermoprobes [34,35]. Limited results on the synthesis of PbF_2_ nanoparticles can be found in the literature. Yb-Er co-doped PbF_2_ nanoparticles have been prepared by two methods, a micellar route and the selective dissolution of the oxide glassy matrix of an oxyfluoride glass–ceramic [35]. In this work, we present a simpler method to produce PbF_2_ nanoparticles that has the potential to permit the growth of core/shell structures based on PbF_2_ cores. The procedure is described in detail in Section 3.

**Figure 2:**
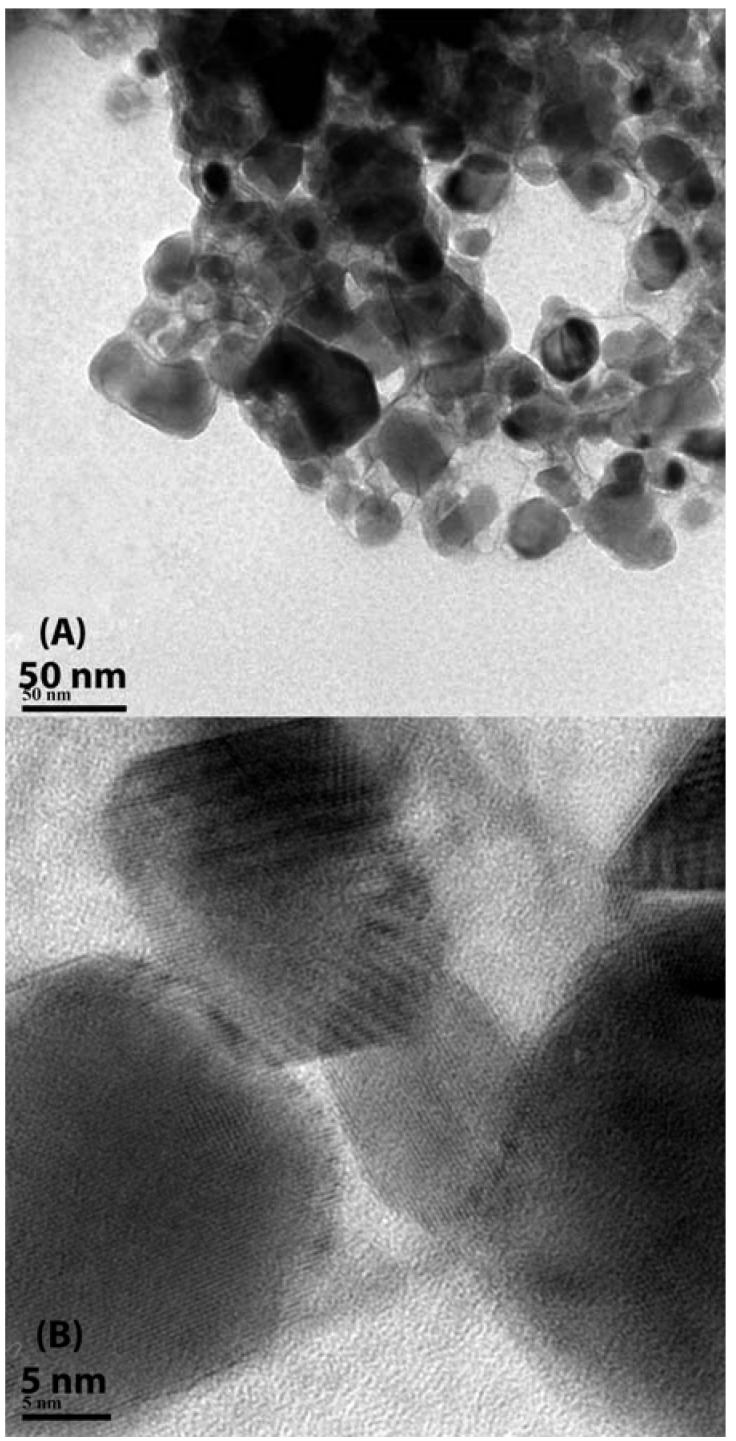
(A) Low magnification, and (B) high magnification TEM images of PbF_2_ nanoparticles.

Basic structural characterization by means of x-ray diffraction (XRD) showed the nanoparticles to have cubic structure in agreement with JCPDF file no. 06–0251. This result is shown in Figure 1, where the PbF_2_ intense diffraction peaks were identified, together with numerous low intensity diffraction peaks from the ligand. Transmission electron microscopy (TEM) results showed agglomerations of polyhedral nanoparticles with sizes ranging from a few to several tens of nanometers (Figure 2A), while high magnification images confirmed the crystalline nature of the nanoparticles through direct observation of crystalline fringes and crystal facets (Figure 2B).

### 2.2. Core/Shell Nanoparticles

In terms of halides core/shell nanoparticles, to date investigations have focused mostly on the lanthanide fluorides [10,29,30,31,32,36,37,38,39]. Core/shell synthesis can be divided in two groups. The first group corresponds to nanoparticles having at least one shell around the original core, where both the core and the shell(s) are composed of the same material either with or without a dopant, e.g., CaF_2_:Eu/CaF_2_. This architecture is hereafter referred to as “self-shelling”. The second group corresponds to core and shell(s) composed of different materials, e.g., LaF_3_/CaF_2_.

Self-shelling has been successfully obtained by our group for LaF_3_, as discussed previously [29,30,31,32], and for CaF_2_, SrF_2_, and BaF_2_ (unpublished results). Here, the results on the group IIA fluorides will be illustrated in detail for CaF_2_:Eu and BaF_2_. BaF_2_ is a fast scintillator with sub-nanosecond emission at 220 nm due to core-valence transitions [40]. This mechanism corresponds to the radiative transition of a fluorine electron in the valence band to a core hole in a Ba ion [41]. Eu-doped CaF_2_ is a relatively bright scintillator, where the Eu^2+^ ions are responsible for strong emission around 420 nm. When in the 3+ valence state, the main emission of Eu ions is at 590 nm [42,43].

**Figure 3:**
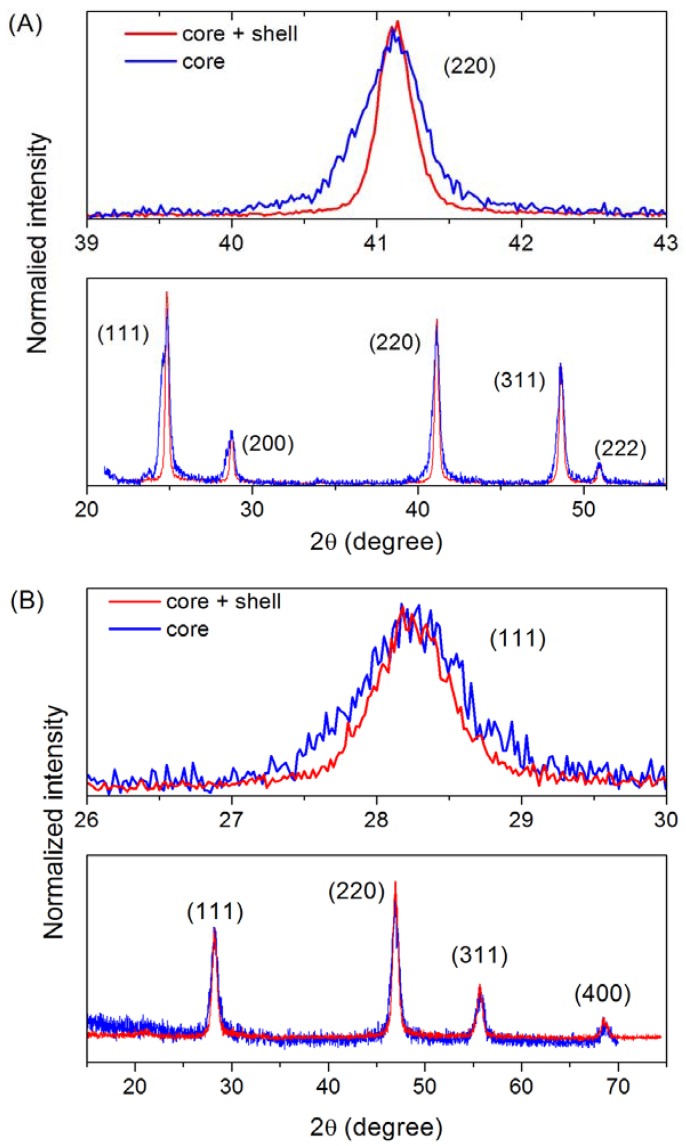
XRD results from core (blue) and core/shell (red) (A) BaF_2_, and (B) CaF_2_:Eu self-shelled nanoparticles. Peak narrowing due to shelling is illustrated for the (220) and (111) planes for BaF_2_/BaF_2_ and CaF_2_:Eu/CaF_2_, respectively.

**Figure 4:**
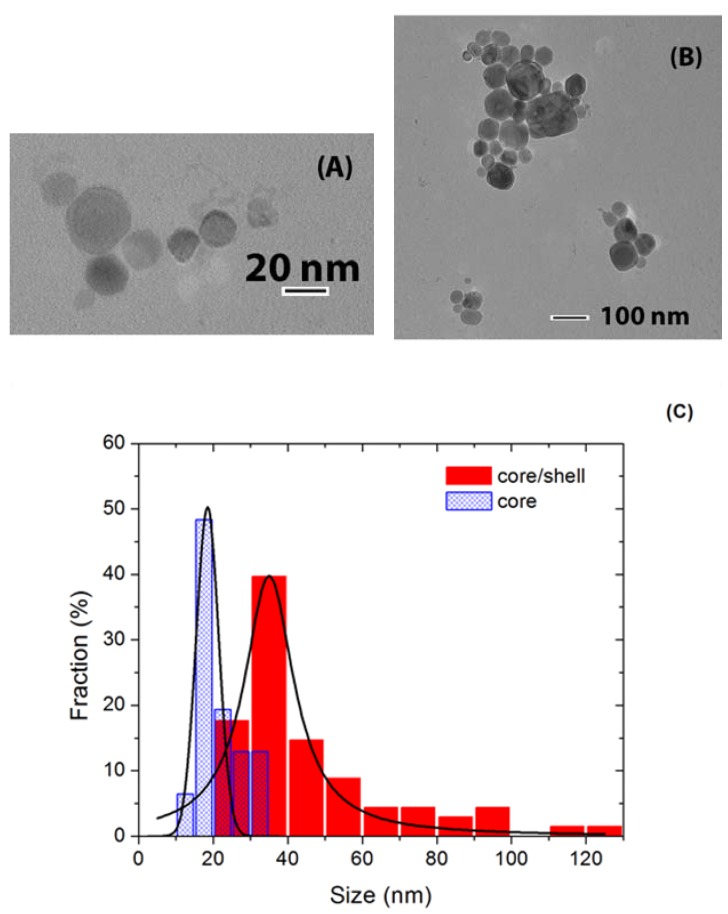
TEM images of (A) core and (B) core/shell BaF_2_/BaF_2_ nanoparticles, and (C) corresponding nanoparticle size histogram.

**Figure 5:**
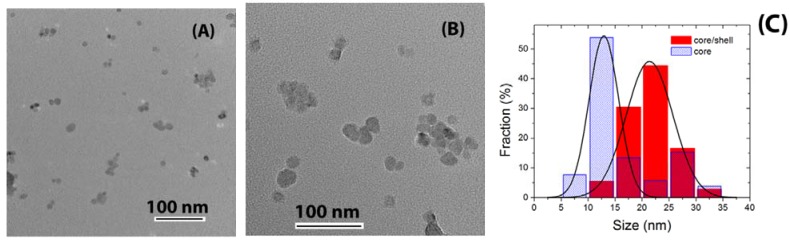
TEM images of (A) core and (B) core/shell CaF_2_:Eu/CaF_2_ nanoparticles, and (C) corresponding nanoparticle size histogram.

The crystallographic structure of these nanoparticles was characterized by means of XRD measurements which revealed a cubic structure in agreement with JCPDF files 35-0816 and 04-0452 for CaF_2_ and BaF_2_, respectively, as shown in Figure 3. Shelling was evaluated by two techniques, XRD and TEM. TEM provides direct imaging of the nanoparticles, and comparison of images of the core and core/shell nanoparticles naturally allows for the verification of successful shelling. Also, analysis of the width of the diffraction peaks can reveal if the average size of the nanoparticles increased after shelling. The combination of these two techniques forms a powerful characterization method since TEM is capable of detecting shelling in individual nanoparticles, while XRD verifies the average behavior of large amounts of nanoparticles. TEM images and size histograms extracted from TEM images are shown in Figure 4 and Figure 5 for BaF_2_/BaF_2_ and CaF_2_:Eu/CaF_2_, respectively. They show the self-shelled particles to have average diameters significantly larger than that of the original cores. For CaF_2_:Eu/CaF_2_, the average diameter increased *ca*. 60%, from 13 ± 3 nm to 21 ± 4 nm, and for BaF_2_/BaF_2_ it doubled, increasing from 18 ± 3 nm to 35 ± 8. These results are supported by the analysis of the width of diffraction peaks of the core (blue) and core/shell (red) nanoparticles, and illustrated in Figure 3A for the (220) diffraction peak of BaF_2_(/BaF_2_), and in Figure 3B for the (111) diffraction peak of CaF_2_:Eu(/CaF_2_). Narrowing of the diffraction peaks confirmed that the average size of the nanoparticles increased.

**Figure 6:**
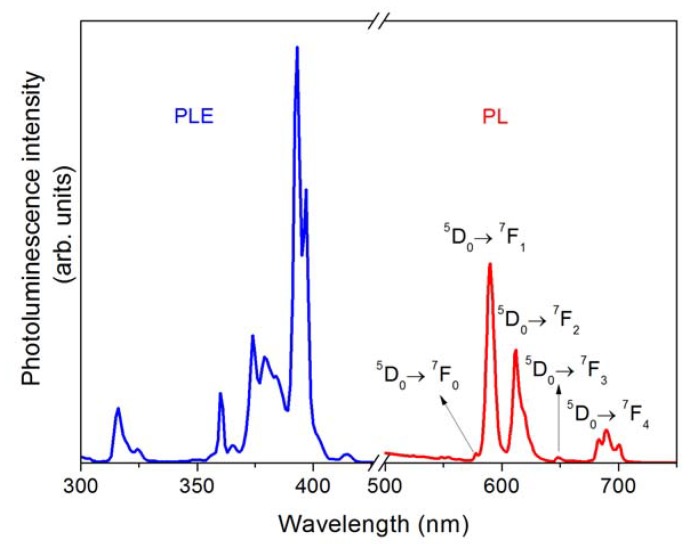
Photoluminescence excitation (PLE) and emission (PL) spectra ascribed to the ^5^D_0_ ↔ ^7^F_J_ (J = 0, 1, 2, 3, and 4) transitions of Eu^3+^ ion in CaF_2_ nanoparticles.

**Figure 7:**
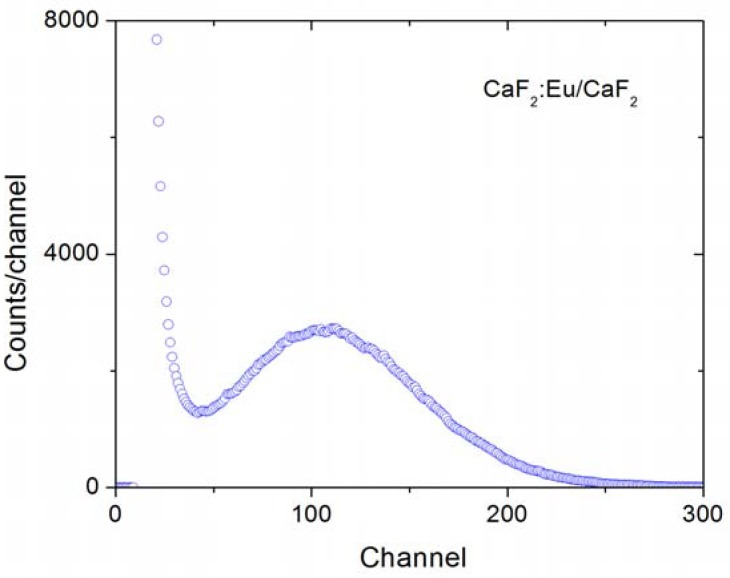
Pulse height distribution measurement of CaF_2_:Eu/CaF_2_ nanoparticles under excitation of ^241^Am source.

Photoluminescence results are shown in Figure 6. Interestingly, Eu ions are incorporated in CaF_2_ nanoparticles in the 3+ state as can be easily identified by the intense emission at 590 nm shown in Figure 6. Further, the sharp excitation (PLE) and emission (PL) peaks can be ascribed to the ^5^D_0_ ↔ ^7^F_J_ (J = 0, 1, 2, 3, and 4) transitions of Eu^3+^ ion. We also note that while the ^5^D_0_ → ^7^F_1_ transition is magnetic dipole allowed, the ^5^D_0_ → ^7^F_2_ transition is allowed by a hypersensitive electric dipole mechanism. The dominance of the first over the later in the observed spectrum indicates that the Eu^3+^ ions are located in a site with inversion symmetry, in agreement with previous results [28]. The scintillation response of these nanoparticles was also investigated under excitation of ^241^Am source by means of pulse height distribution measurements. Figure 7 shows a typical result where a well-defined photopeak from CaF_2_:Eu/CaF_2_ nanoparticles was observed, confirming the potential of these nanoparticles as scintillators.

While self-shelling is expected to occur due to the natural chemical affinity between the core and shell, in the case of shelling of a core by a different compound the result from the competition between shelling and nucleation and growth of new nanoparticles is more difficult to predict. To date, among the fluorides, shelling of a core with a different compound has been reported for CeF_3_:Tb core by a LaF_3_ shell [37], and for EuF_3_ core by a Eu_0.65_Gd_0.35_F_3_ shell [38]. In this work, we discuss our attempts to shell CaF_2_ nanoparticles with LaF_3_, and to shell LaF_3_ nanoparticles with CaF_2_.

**Figure 8:**
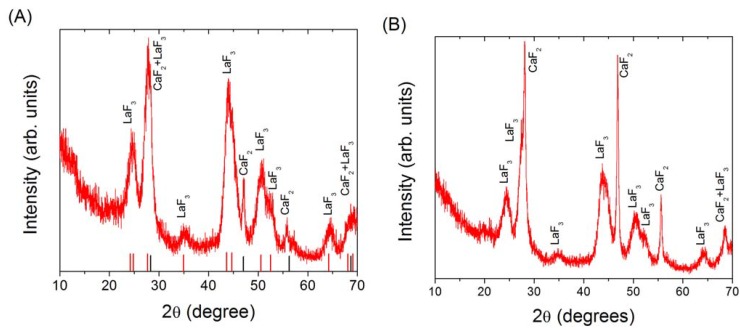
(A) XRD of LaF_3_/CaF_2_ core/shell nanoparticles, and (B) XRD results of the attempt to obtain CaF_2_/LaF_3_ core/shell nanoparticles. The JCPDF files 35-0816 for cubic CaF_2_ (black lines), and 32-0483 for hexagonal LaF_3_ (red lines) are also shown.

**Figure 9:**
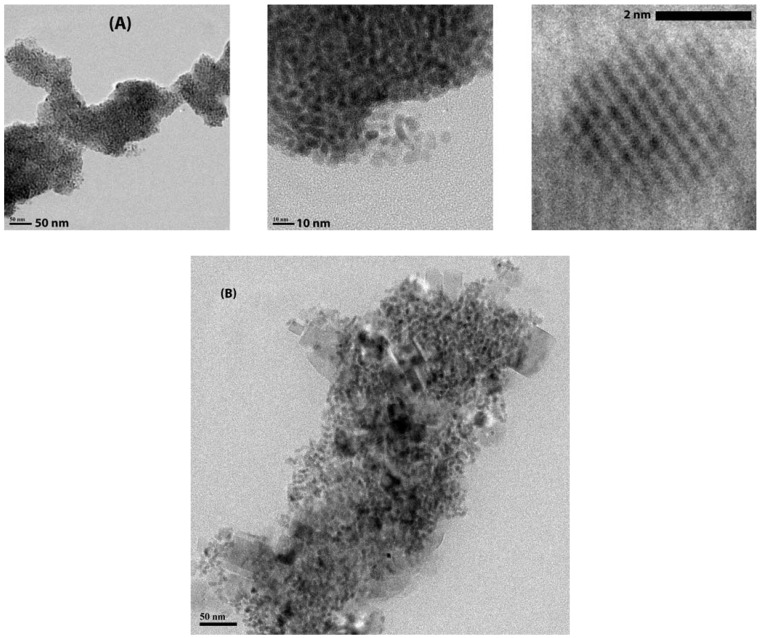
(A) TEM images with progressing magnification of the LaF_3_/CaF_2_ system. From left to right, it is possible to observe large agglomerates of LaF_3_ nanoparticles embedded in a matrix of CaF_2_ (left), forming a nanocomposite (middle) with well-defined LaF_3_ nanoparticles (right). (B) TEM image of the CaF_2_/LaF_3_ system, where LaF_3_ and CaF_2_ nanoparticles agglomerated forming a mixture without any architectural hierarchy.

LaF_3_/CaF_2_ and CaF_2_/LaF_3_ core/shell nanoparticles where synthesized similarly to the descriptions above for these individual materials. XRD measurements shown in Figure 8 revealed the presence of cubic CaF_2_ (JCPDF 35-0816) and hexagonal LaF_3_ (JCPDF 32-0483) in both samples, confirming the successful synthesis of both fluorides. On the other hand, TEM imaging revealed very different results after shelling. Figure 9A illustrates the results of our attempt to shell LaF_3_ with CaF_2_, where individual sub 5 nm LaF_3_ nanoparticles can be easily identified embedded in a matrix of CaF_2_ forming large agglomerates in a nanocomposite fashion. While shelling was achieved, at this stage it was not possible to obtain isolated LaF_3_/CaF_2_ core/shell nanoparticles. As for the attempt to synthesize CaF_2_/LaF_3_ core/shell nanoparticles, while both phases were successfully synthesized (Figure 8B), TEM imaging revealed that shelling did not occur, with particles of both materials agglomerating to each other without any structural hierarchy, forming a mixture (Figure 9B).

## 3. Experimental Section

The synthesis of nanoparticles was carried out by means of modified solution precipitation methods described in detail for each compound. Briefly, they consist of mixing a NH_4_F/ADDP (ammonium di-*n*-octadecyldithiophosphate) solution in 1:1 ethanol (Acros Organic, 99.5%):water together with nitrate solutions of the metals corresponding to the host and dopants. ADDP was used as ligand to allow for the formation of shells, while simultaneously avoiding particle aggregation [20]. ADDP was produced at our laboratories following the procedure described in ref. [20]; water was purified using a Nanopure Diamond purification system from Barnstead International. In this work, the following notation for core/shell was adopted: M1F_n_:RE1/M2F_m_:RE2, where compound M1F_n_ doped with rare earth “1” forms the core that has a shell of compound M2F_m_ doped with rare earth “2”.

The synthesis of LaF_3_ nanoparticles made use of a solution of 2.46 g ADDP and 16 mmol NH_4_F (Acros Organic, >98%) in 140 mL 1:1 ethanol:water heated at 75 °C, and another solution of 5.33 mmol La(NO_3_)_3_ (Aldrich, 99.99%) dissolved in 4 mL H_2_O was added dropwise to the fluorinated solution to form the core. Ligand ADDP has the dual function of avoiding particle agglomeration and allowing the growth of shells around the core. After stirring for 10 min., the first shell was grown by alternating addition in 10 parts of a 2 mL aqueous NH_4_F (12 mmol) solution and a 2 mL aqueous solution with x mmol RE(NO_3_)_3_, with RE = Tb and Eu (Alfa Aesar, 99.9%), and 4-x mmol La(NO_3_)_3_, where x determines the RE dopant concentration [29].

Similarly to the synthesis of LaF_3_, we make use of a solution of 2.46 g ADDP and 13.6 mmol NH_4_F (Acros Organic, >98%) in 280 mL 1:1 ethanol:water heated at 75 °C, and another solution of 7.97 mmol Pb(NO_3_)_2_ (Fisher, reagent grade) dissolved in 8 mL H_2_O dropwise added to the fluorinated solution to form the PbF_2_ nanoparticles. The use of higher volumes of these solutions when compared to the case of LaF_3_ is to compensate for the higher viscosity of the final mixture.

The synthesis of CaF_2_:Eu/CaF_2_ and BaF_2_/BaF_2_ self-shelled core/shell nanoparticles takes advantage of the organic ligand ADDP to achieve shelling and to avoid agglomeration, as already demonstrated for other materials [10,20,29,30,31,32,36]. The procedure consists of preparing a fluorinated solution in 35 mL of 1:1 ethanol:water at 75 °C from 13.6 mmol NH_4_F (Acros Organic, >98%) together with 2.46 g ADDP, and another solution of the host and dopant metal nitrates (Ca(NO_3_)_2_∙4H_2_O (Fisher, 99.9%), Eu(NO_3_)_3_∙6H_2_O (Alfa Aesar, 99.9%), Ba(NO_3_)_2_ (Fisher, reagent grade), and Ce(NO_3_)_3_∙6H_2_O (Acros Organic, 99.5%)) in water, followed by drop-wise addition of the nitrate solution into the fluorinated solution while stirring to form the cores in suspension. After addition of the nitrate solution, stirring continues for 10 min., and then additional fluorinated and nitrate solutions are alternatively added in 10 parts to the core suspension to form a shell. The final solution is stirred for 10 min., and then cooled down to room temperature. Table 1 summarizes the specific conditions for the precursor solutions. Further, the precipitates were cleaned by washing in ethanol and water, followed by dispersion in dichloromethane (Acros, anhydrous, AcroSeal (TM), 99.9%) and precipitation with the addition of 20 mL of ethanol. The resultant powder was dried for 2 days over P_2_O_5_ in a desiccator. The nanoparticles could be dispersed in tetrahydrofuran (Acros, anhydrous, AcroSeal (TM), 99.9%) for characterization and testing.

**Table 1:** Synthesis conditions for self-shelling and 3 mol % RE doping.

Core/Shell	Precursor solution
CaF_2_:Eu/CaF_2_	Core: 1.94 mmol Ca(NO_3_)_2_*4H_2_O + 0.06 mmol Eu(NO_3_)_3_*6H_2_O in 2 mL H_2_O
	Shell: 2 mmol Ca(NO_3_)_2_*4H_2_O in 2 mL H_2_O and 3.41 mmol NH_4_F in 2 mL H_2_O
BaF_2_:Ce/BaF_2_	Core: 1.94 mmol Ba(NO_3_)_2_ + 0.06 mmol Ce(NO_3_)_3_*6H_2_O in 5.81 mL H_2_O
	Shell: 2 mmol Ba(NO_3_)_2_ in 5.99 mL H_2_O and 3.41 mmol NH_4_F in 2 mL H_2_O

Morphological and particle size analysis was carried out by means of TEM using Hitachi microscopes 9500 with 300 kV acceleration voltage, HD2000 with 200 kV acceleration voltage, and H7600T with 120 kV acceleration voltage.

Structural characterization was carried out by means of XRD measurements using a Scintag XDS 4000 diffractometer equipped with a Cu Kα source aiming at phase identification and crystallite size estimation based on the Debye-Scherrer method.

Photoluminescence measurements were carried out using a Horiba Jobin-Yvon Fluorolog-322 spectrofluorimeter with a double grating configuration in ambient conditions.

Scintillation characterization was carried out in terms of pulse height distribution measurements using a Hidex Triathler scintillation counter and a 1 μCi ^241^Am source that emits mostly 5.5 MeV alpha particles and 60 keV gamma-rays.

## 4. Conclusions

The synthesis, characterization and application of RE-doped metal fluoride nanoparticles was presented and discussed, focusing on the results obtained by our group, particularly on core/(multi-) shell nanoparticles. Many systems were investigated, including heavy metal fluorides LaF_3_ and PbF_2_, and group IIA fluorides such as CaF_2_ and BaF_2_ in self-shelled and mixed compounds core/shell architectures. It is possible to conclude from the above results that while significant control of materials characteristics and luminescent properties can be achieved through core/shell architectures, the state-of-the-art on the synthesis of core/shell nanoparticles is in its infancy, and successful outcome conditions are still determined using an Edisonian approach.

The investigation of nanoscale materials opened up a new dimension in materials science. Materials can now be produced and modified at the atomic scale, and an unparallel control of properties and functionalities has been achieved or is, at least, envisioned. In nearly 20 years, a vast amount of discoveries were made, generating great excitement within the scientific community and captivating the general public on the technological potential and future development of nanomaterials. While the full impact and risks of nanotechnology have not been evaluated in depth, as Sass puts it, “*history is an alloy of all the materials that we have invented or discovered*” [44] and nanoparticles will certainly and increasingly contribute to underpin mankind’s endeavors.

## References

[1] Murr L.E. (2007). Nanoparticulate materials in antiquity: The good, the bad and the ugly. Microsc. Microanal..

[2] Vaughan A. (2008). Raman nanotechnology−the Lycurgus Cup. IEEE Elec. Insul. Mag..

[3] Reibold M., Paufler P., Levin A.A., Kochmann W., Pätzke N., Meyer D.C. (2006). Carbon nanotubes in an ancient Damascus sabre. Nature.

[4] Feynman R. (1992). There’s plenty of room at the bottom. J. Microelectromechanical Syst..

[5] Birringer R., Gleiter H., Klein H.-P., Marquardt P. (1984). Nanocrystalline materials an approach to a novel solid structure with gas-like disorder?. Phys. Lett. A.

[6] Edelstein A.S., Cammarata R.C. (1996). Nanomaterials: Synthesis, Properties and Applications.

[7] Canham L.T. (1990). Silicon quantum wire array fabrication by electrochemical and chemical dissolution of wafers. Appl. Phys. Lett..

[8] Chander H. (2005). Development of nanophosphors−A review. Mater. Sci. Eng. R.

[9] Cooke D.W., Lee J.-K., Bennett B.L., Groves J.R., Jacobsohn L.G., McKigney E.A., Muenchausen R.E., Nastasi M., Sickafus K.E., Tang M., Valdez J.A., Kim J.-Y., Hong K.S. (2006). Luminescent properties and reduced dimensional behavior of hydrothermally prepared Y_2_SiO_5_:Ce nanophosphors. Appl. Phys. Lett..

[10] Stouwdam J.W., van Veggel C.J.M. (2004). Improvement in the luminescence properties and processability of LaF_3_/Ln and LaPO_4_/Ln nanoparticles by surface modification. Langmuir.

[11] Kömpe K., Leehmann O., Haase M. (2006). Spectroscopic distinction of surface and volume ions in cerium(III)- and terbium(III)-containing core and core/shell nanoparticles. Chem. Mater..

[12] Lecoq P., Annenkov A., Gektin A., Korzhik M. (2006). Inorganic Scintillators for Detector Systems: Physical Principles and Crystal Engineering.

[13] Klocek P. (1991). Handbook of Infrared Optical Materials.

[14] Henderson B., Imbusch G.F. (1989). Optical Spectroscopy of Inorganic Solids.

[15] Su L., Wang C., Chai L., Xu X., Zhao G. (2005). Low-threshold diode-pumped Yb^3+^,Na^+^:CaF_2_ self-Q-switched laser. Opt. Exp..

[16] Qiu S.Q., Dong J.X., Chen G.X. (1999). Tribological properties of CeF_3_ nanoparticles as additives in lubricating oils. Wear.

[17] Qiu S.Q., Dong J.X., Chen G.X. (2000). Synthesis of CeF_3_ nanoparticles from water-in-oil microemulsions. Powder Technol..

[18] Zhou J.F., Wu Z.S., Zhang Z.J., Liu W.M., Dang H.X. (2001). Study on an antiwear and extreme pressure additive of surface coated LaF_3_ nanoparticles in liquid paraffin. Wear.

[19] Stouwdam J.W., van Veggel F.C.J.M. (2002). Near-infrared emission of redispersible Er^3+^, Nd^3+^, and Ho^3+^ doped LaF_3_ nanoparticles. Nano Lett..

[20] Stouwdam J.W., Hebbink G.A., Huskens J., van Veggel F.C.J.M. (2003). Lanthanide-doped nanoparticle with excellent luminescent properties in organic media. Chem. Mater..

[21] Eiden-Assmann S., Maret G. (2004). CeF_3_ nanoparticles: Synthesis and characterization. Mat. Res. Bull..

[22] Lian H., Zhang M., Liu J., Ye Z., Yan J., Shi C. (2004). Synthesis and spectral properties of lutetium-doped CeF_3_ nanoparticles. Chem. Phys. Lett..

[23] Bender C.M., Burlitch J.M., Barber D., Pollock C. (2000). Synthesis and fluorescence of neodymium-doped barium fluoride nanoparticles. Chem. Mater..

[24] Sun X.M., Li Y.D. (2003). Size-controllable luminescent single crystal CaF_2_ nanocubes. Chem. Commun..

[25] Hua R.N., Zang C.Y., Sha C., Xie D.M., Shi C.S. (2003). Synthesis of barium fluoride nanoparticles from microemulsion. Nanotechnol..

[26] Lian H.Z., Liu J., Ye Z.R., Shi C.S. (2004). Synthesis and photoluminescence properties of erbium-doped BaF_2_ nanoparticles. Chem. Phys. Lett..

[27] Grass R.N., Stark W.J. (2005). Flame synthesis of calcium-, strontium-, barium fluoride nanoparticles and sodium chloride. Chem. Commun..

[28] Wang F., Fan X.P., Pi D.P., Wang M.Q. (2005). Synthesis and luminescence behavior of Eu^3+^-doped CaF_2_ nanoparticles. Solid State Commun..

[29] DiMaio J.R., Kokuoz B., James T.L., Ballato J. (2007). Structural determination of light-emitting inorganic nanoparticles with complex core/shell architectures. Adv. Mater..

[30] DiMaio J.R., Sabatier C., Kokuoz B., Ballato J. (2008). Controlling energy transfer between multiple dopants within a single nanoparticle. Proc. Natl. Acad. Sci. USA.

[31] DiMaio J.R., Kokuoz B., Ballato J. (2006). White light emissions through down-conversion of rare-earth doped LaF_3_ nanoparticles. Opt. Exp..

[32] DiMaio J.R., Kokuoz B., James T.L., Harkey T., Monofsky D., Ballato J. (2008). Photoluminescent characterization of atomic diffusion in core-shell nanoparticles. Opt. Exp..

[33] Anderson D.A., Kierstead J.A., Lecoq P., Stoll S., Woody C.L. (1994). A search for scintillation in doped and orthorhombic lead fluoride. Nuc. Instrum. Methods Phys. Res. A.

[34] Aigouy L., Tessier G., Mortier M., Charlot B. (2005). Scanning thermal imaging of microelectronic circuits with fluorescent nanoprobe. Appl. Phys. Lett..

[35] Lab´eguerie J., Dantelle G., Gredin P., Mortier M. (2008). Luminescence properties of PbF_2_:Yb–Er nanoparticles synthesized by two different original routes. J. Alloys Comp..

[36] Sudarsan V., van Veggel F.C.J.M., Herring R.A., Raudsepp M. (2005). Surface Eu^3+^ ions are different than “bulk” Eu^3+^ ions crystalline doped LaF_3_ nanoparticles. J. Mater. Chem..

[37] Wang Z.L., Quan Z.W., Jia P.Y., Lin C.K., Luo Y., Chen Y., Fang J., Zhou W., O’Connor C.J., Lin J. (2006). A facile synthesis and photoluminescent properties of redispersible CeF_3_, CeF_3_:Tb^3+^, and CeF_3_:Tb^3+^/LaF_3_ (core/shell) nanoparticles. Chem. Mater..

[38] Lezhnina M.M., Justel T., Katler H., Wiechert D.U., Kynast U.H. (2006). Efficient luminescence from rare-earth fluoride nanoparticles with optically functional shells. Adv. Func. Mater..

[39] Mai H.X., Zhang Y.W., Sun L.D., Yan C.H. (2007). Highly efficient multicolor up-conversion emissions and their mechanisms of monodisperse NaYF_4_:Yb, Er core and core/shell-structured nanocrystals. J. Phys. Chem. C.

[40] Laval M., Moszynski M., Allemand R., Cormoreche E., Guinet P., Odru R., Vacher J. (1983). Barium fluoride inorganic scintillator for subnanosecond timing. Nuc. Instrum. Methods Phys. Res..

[41] Rodnyi P.A. (2004). Core-valence luminescence in scintillators. Rad. Meas..

[42] Rodnyi P.A., Khadro A.K., Voloshinovskii A.S., Stryganyuk G.B. (2007). Europium luminescence in fluorite upon high-energy excitation. Opt. Spec..

[43] Pandey C., Dhopte S.M., Muthal P.L., Kondawar V.K., Moharil S.V. (2007). Eu^3+^ ↔ Eu^2+^ redox reactions in bulk and nano CaF_2_:Eu. Rad. Eff. Defect. Solid..

[44] Sass L.S. (1998). The Substance of Civilization.

